# Topographical influence of electrospun basement membrane mimics on formation of cellular monolayer

**DOI:** 10.1038/s41598-023-34934-x

**Published:** 2023-05-24

**Authors:** Puja Jain, Rahul Rimal, Martin Möller, Smriti Singh

**Affiliations:** 1 DWI-Leibniz Institute for Interactive Materials, Forckenbeckstr. 50, 52074 Aachen, Germany; 2 Max Planck Institute for Medical Research (MPImF), Jahnstrasse 29, 69120 Heidelberg, Germany

**Keywords:** Cell biology, Materials science

## Abstract

Functional unit of many organs like lung, kidney, intestine, and eye have their endothelial and epithelial monolayers physically separated by a specialized extracellular matrix called the basement membrane. The intricate and complex topography of this matrix influences cell function, behavior and overall homeostasis. In vitro barrier function replication of such organs requires mimicking of these native features on an artificial scaffold system. Apart from chemical and mechanical features, the choice of nano-scale topography of the artificial scaffold is integral, however its influence on monolayer barrier formation is unclear. Though studies have reported improved single cell adhesion and proliferation in presence of pores or pitted topology, corresponding influence on confluent monolayer formation is not well reported. In this work, basement membrane mimic with secondary topographical cues is developed and its influence on single cells and their monolayers is investigated. We show that single cells cultured on fibers with secondary cues form stronger focal adhesions and undergo increased proliferation. Counterintuitively, absence of secondary cues promoted stronger cell–cell interaction in endothelial monolayers and promoted formation of integral tight barriers in alveolar epithelial monolayers. Overall, this work highlights the importance of choice of scaffold topology to develop basement barrier function in in vitro models.

## Introduction

Cells are embedded in a complex and dynamic microenvironment that exhibit unique biochemical and biomechanical properties at varying locations in the human body^1^. One such complex and specialized microenvironment is termed the basement membrane (BM)^2^. This specialized matrix is associated with important roles of maintaining, separating, and supporting the overlying cell layers, as well as incorporation of various growth factors and signaling molecules to maintain tissue homeostasis^3–6^. The complex organization of protein constituents including collagen IV and laminin impart a 3D fibrous architecture to the dense BM scaffold in vivo^7^. Measurement techniques such as atomic force microscopy (AFM) and electron microscopy have revealed intricate structures of the BM including pores in the range of 50 nm and microstructural fibrous arrangements with elevated regions^4,8–11^. It is well known that cells can sense such nano- and micro-topographical cues and exhibit variable responses. These include changes in proliferation, morphology, migration, wound healing, as well as cell alignment^12–17^. However, the ultrastructure of BM is not entirely understood and new findings are constantly being made^3,18–21^.

Prior cell-material interaction studies have mainly focused on the biological and chemical properties of the BM and their influence on tissue function and cellular composition^7,22–25^. These studies include cell interaction with different combinations of extracellular matrix (ECM) components such as collagen, laminin as well as the use of natural BM such as Matrigel or decellularized ECM^5,23–25^. Additionally, in terms of structural and mechanical properties, studies have mainly focused on the thickness and stiffness aspect of the BM which is known to change during the different stages of growth, age and disease progression^17,21,26^. Existing in vitro BM scaffolds have been engineered and designed to achieve thickness and stiffness close to the in vivo conditions including ultra-thin membranes that resemble the BM of the alveolar-capillary barrier^27–30^. However, only a few studies focus on the topographical influence of the BM on cellular behavior and response^12,31^, where majority of the BM scaffolds are 2D patterned polymeric substrates that employ photolithography, micro-contact printing, molding, and chemical etching^32,33^. These techniques are valuable to recapitulate topographical features such as grooves, pillars and pores known to enhance cellular adhesion, and proliferation, but only a few studies report this effect using fibrous substrates^34–37^. However, studies conducted by Chen et al. report that cellular behavior on fibrillar meshes is more physiological compared to widely used flat hydrogel substrates^38,39^. Interestingly, existing studies using fibrous substrates have mainly considered influence of directional cues on endothelial and nerve cell alignment by employing aligned or randomly distributed fibers^40–42^. However, the influence of secondary topographical cues on such fibrous scaffolds is not well reported. Although, engineered fibrous substrates with secondary surface cues such as grooves and pits have been designed to investigate single cell responses, they are less well studied using cell monolayers to gain insight on cell–cell junction or barrier formation like in pulmonary air–liquid interface (ALI), blood retinal barrier (BRB) or the blood–brain barrier (BBB)^16,40,43,44^. Moreover, in vivo cells are not present as singular components rather they are present as a single layer of epithelial, endothelial or other stromal cells supported on a BM^26^. This is further augmented by a recent study from Ippolito et al. which emphasizes that in vivo like mechanical and biological function of the tissue can only be attained by suitable morphological and cellular arrangement of cells in a monolayer^45^. This highlights the importance of scaffold design to achieve functional monolayers.

However, there is limited knowledge on cell monolayer formation and response on engineered fibrous scaffolds with secondary topographical cues. Whether secondary cues are an advantage as shown by single cell experiments has not been reported for monolayers^46^.

Techniques to fabricate fibrous substrates with secondary topographical features include 3D printing, melt spinning and dry spinning^47–49^. Among these, electrospinning is a simple and versatile technique that allows fabrication of synthetic non-woven meshes that resemble the disordered structure of the natural BM^50^. In addition, the choice of solvent systems and optimum electrospinning parameters also enable the formation of topographical cues on the fiber surface^50^. Furthermore, the fiber meshes can be experimentally tuned to exhibit similar fiber density and mesh pore size. This negates the influence of overall geometry on cells and cells respond predominantly to the secondary nano-features on the fiber surface. Very few cell-material interaction studies have been conducted where the nano-scale cues presented on a fibrous BM mimic are employed to examine the response and behavior of cells like epithelial and endothelial cells which are predominantly present in most of the physiological barriers^8,18,27,28^. Thus, such electrospun fibrous scaffolds provide ideal platforms to expand our understanding into the less explored subject of influence of secondary nano-scale features on the development and function of barrier models.

In this work we take the example of pulmonary alveolar-capillary barrier formed by confluent epithelial and endothelial cell and investigate the influence of secondary topographical cues presented on non-woven BM mimics, towards formation of functional monolayers. Electrospinning with specific solvent system was employed to fabricate smooth and nano-pores as secondary topographical cues on non-woven PCL fiber meshes with similar fiber diameter and mesh pore size. We tested the single cell morphology, proliferation, monolayer barrier formation, and cell–cell tight junctions of cells cultured on the non-woven meshes with and without topological cues. Moreover, single cell analysis on focal adhesion formation including vinculin and actin filament organization was conducted to highlight the influence of the secondary nano-scale features on cell adhesion and cell spreading. Furthermore, the formation of stable alveolar epithelial barrier was investigated by analysis of size and number of lamellar bodies as well as formation of micro-villi which is an indicator of alveolar type II cells. This work emphasizes the need to differentiate the influence of secondary nano-cues on single cell and organ level response. Our study concludes that although secondary nano-pores on fiber surfaces presented as meshes facilitate cellular attachment and proliferation, such cues are unfavorable in the formation of optimal alveolar barrier models.

## Results and discussion

### Fabrication of porous and smooth PCL fibers

**Figure 1:**
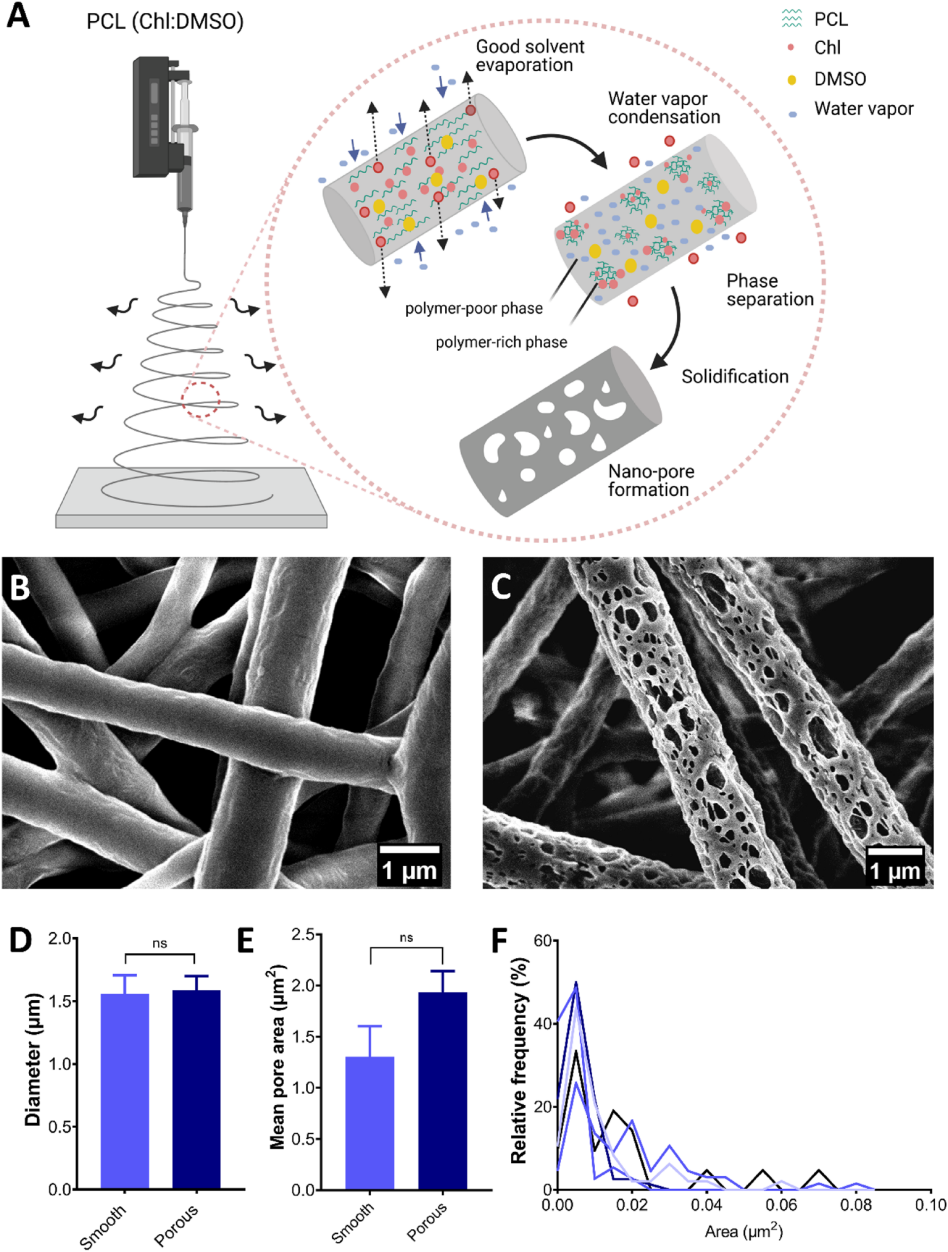
(**A**) Schematic representation of the mechanism of pore formation on fiber surface during the electrospinning process. The secondary nano-structure on the microfiber is obtained through a process of phase separation when an optimum ratio of good and bad solvent is used during solution preparation. (**B**) represents homogenous non-woven electron micrograph of smooth surface fibers and (**C**) porous surface fibers. The graphs represent fabrication of non-woven meshes with (**D**) similar fiber diameter, and (**E**) mean mesh pore area of both fiber types, (**F**) represents pore area distribution of the secondary surface pores present on the fiber surface shown in c; n = 5.

Electrospinning is a versatile technique that can be employed to generate fibers with secondary features such as nano-pores. Commonly used methods to generate pores are by phase separation or spinning in a humid environment, where the evaporation of water molecules from the surface of the fiber generates pores. Phase separation is a method where the polymer solution separates into polymer-rich and polymer-poor phase, which contributes to the formation of the matrix and pores respectively (Fig. 1A). Here, we utilized the non-solvent induced phase separation phenomenon, where 12.5% w/v PCL was dissolved in a binary solvent system comprising good solvent (chloroform) and a non-solvent (dimethyl sulfoxide, DMSO) with higher boiling point at a 90/10 (V/V) ratio. The presence of DMSO initiates a thermodynamic instability and leads to phase separation, where the chloroform evaporates faster than the DMSO. This presence of the non-solvent DMSO leads to formation of pores on the fiber surface (Fig. 1C). The electrospinning properties of 21 kV, 17 cm (distance between collector and spinneret), 0.75 mL/h (flow rate) resulted in formation of fibrous non-woven meshes with pores on fiber surfaces. Similarly, to achieve fibers with a comparable diameter and smooth surface (Fig. 1B), a combination of chloroform and methanol at 50/50 (V/V) ratio were used to dissolve 12.5% w/v PCL, where both chloroform and methanol are good solvents of PCL.

Additionally, optimization of spinning parameters was essential in obtaining similar fiber diameter on both the smooth and porous fiber surfaces. The electrospinning properties of 21 kV, 10 cm (distance between collector and spinneret), and flow rate of 1 mL/h resulted in smooth non-woven fibrous meshes.

### Fiber characterization

Scaffold properties are integral in the field of tissue engineering as they significantly influence cell behaviour and response. Thus, the electrospun meshes obtained with and without pores on the fiber surface were investigated in terms of fiber diameter; mesh pore area; pore size; water contact angle; and tensile properties. Optimum spinning parameters led to formation of porous fibres (1.58 µm ± 0.11 µm) and smooth fibres (1.56 µm ± 0.14 µm), Fig. 1D with similar micron range fiber diameters inferred from diameter plugin of ImageJ software. The mean area of interfiber pores was determined and showed no significant difference between porous fibers (1.93 µm^2^ ± 0.20 µm^2^) and smooth fibers (1.30 µm^2^ ± 0.29 µm^2^), Fig. 1E. Both smooth and porous surface fibers had similar fiber diameter and interfiber distance. This excludes the influence of fiber diameter and interfiber distance on cell behaviour and response. Therefore, the absence and presence of nano-pores on the fiber surfaces is considered to have a major influence on cell response. The secondary porous cues are non-uniform in size and of nano-scale nature with an approximate pore area of 0.01 µm^2^, Fig. 1F. This is in range with nano-pits (0.01–0.18 µm^2^) fabricated by Lim et al*.* which stimulated osteoblastic cell adhesion^51^. The loss of surface area due to pore formation can be calculated indirectly using image J. Based on calculations achieved from randomly selected fibers, it was found that a total of 17.3% ± 3.9% of fiber surface area is lost when compared to the smooth surface fibers. Both smooth and porous fiber meshes have similar interfiber porosity and fiber diameter, however the cells on porous fibers have an average of ~ 20% less surface area compared to cells on smooth fibers. This difference in surface area available to cells could contribute to the observed differences in cell behaviour. Furthermore, the mechanical properties of the porous fiber meshes are impacted by the pores present on its surface. The non-solvent induced phase separation by employing DMSO (bad solvent) and chloroform (good solvent) lead to the formation of pores with an area of 0.018 µm^2^ and an equivalent pore diameter of 64–87 nm. In order to assess the diffusion of molecules across the smooth and porous meshes, passage of FITC-dextran (70 kDa) was conducted to examine if the presence of surface fiber pores affect the passage of molecules. It was observed that the diffusion of FITC-dextran from the apical side to the basal side of the meshes was slightly more (as observed by the fluorescent intensity values of solution taken from the basal side) across the smooth meshes as compared to the porous meshes. However, this was not significantly more when compared to the porous meshes, as seen in Supplement figure S2. This difference in the diffusion of dyes has also been previously observed by Wu et al^76^, where the porous fibers lead to adsorbance or entrapment of the dye molecules in the nano-pores, which leads to slight reduction in molecules that pass across the meshes. However, there is no significant difference in passage of molecules across the smooth and porous meshes as they share a similar interfiber distance. Additionally, water contact angle measured by sessile drop method did not show significant difference between the smooth (125.88° ± 3.46°) and porous fiber surfaces (131.73° ± 3.34°) (Supplement figure S1). The wetting properties of the PCL fibers, which are similar in terms of fiber diameter and inter-fiber pore area, indicated hydrophobic nature where the effect of secondary nano-scale features were negligible and did not alter the hydrophobic properties of the non-woven mesh. Elastic modulus of a substrate is also known to influence cell response and was thus characterized by tensile measurements. The non-woven smooth fiber meshes displayed a higher tensile modulus (15.73 MPa ± 3.55 MPa) compared to the porous fiber meshes (5.47 MPa ± 1.72 MPa), as seen in Supplement figure S3. The reduced modulus of the porous fiber surface is expected and can be explained due to presence of surface pores as pre-existing fracture points which enable faster breakage with lower applied forces.

### Single cell analysis and cell proliferation

The fabricated non-woven meshes with and without secondary nano-scale features were used to understand the single cell morphology, focal adhesion, and proliferation of both endothelial (HUVEC) and alveolar epithelial cells (H441). HUVECs and NCI-H441 cells are commonly used as co-cultures to fabricate in vitro alveolar-capillary models. NCI-H441 cell lines resemble the alveolar type II cells and are characterized by high barrier integrity and surfactant production and are often used to replace primary alveolar epithelial cells which are difficult to maintain during long term culture conditions. The presence of secondary nano- pores contribute to surface roughness when compared to the smooth surface fibers. Earlier studies have found that surface roughness affects cell behaviour regardless of the cell type and matrix materials. Presence of nano-roughness is known to have a positive effect on cell adhesion and growth, which is indicated by enhanced endothelial cell growth and adhesion on rough surfaces. Cells sense their microenvironment by membrane protrusions such as lamellipodia and filopodia in the initial stages of cell-surface interaction. Once the cell spreads, they form mature focal adhesion sites including vinculin where clustered integrins interact with the actin cytoskeleton and the external environment. In the case of porous surface fiber meshes, the cells extend their filopodia to feel the surface pores but do not seem to penetrate the surface pores. This is expected as the diameter of filopodia (100–300 nm)^1,2^ is larger than the surface pores (64–87 nm), however, the cells are observed to interact with the surface pores via membrane extensions (Supplement figure S4).

**Figure 2:**
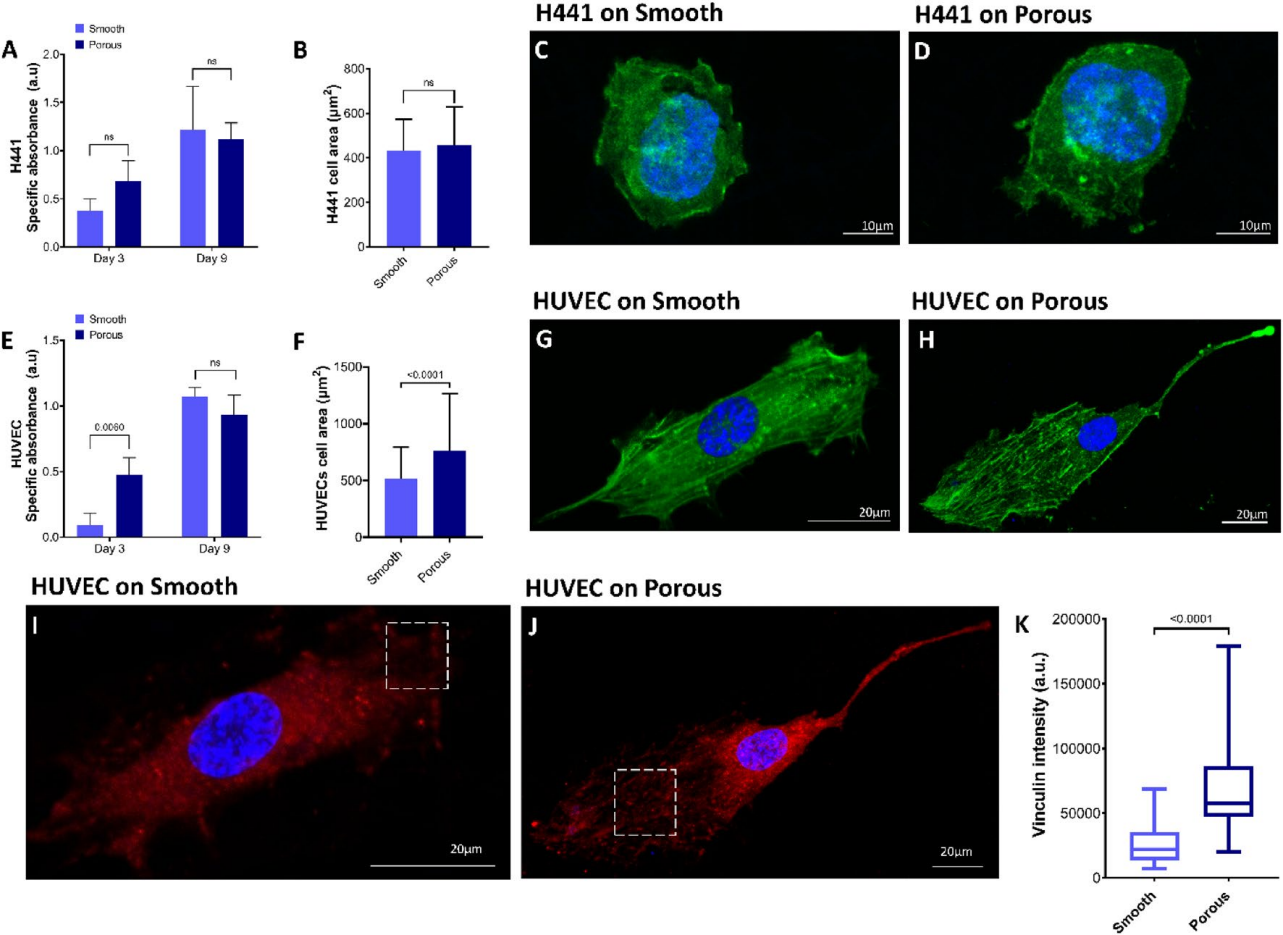
Cell proliferation was analyzed using XTT assay for (**A**) H441 epithelial cells and (**E**) HUVEC endothelial cells on smooth and porous fiber surfaces on day 3 and 9, where proliferation of cells was higher on porous surface fibers on day 3 but no difference was observed for longer culture period day 9; n = 3. Single cell area was investigated using ImageJ where (**B**) H441 cells that did not show significant difference but (**F**) HUVEC displayed a larger cell area on porous surface fibers; n = 115. Confocal images where (**C**), (**D**) H441 cells and (**G**), (**H**) HUVEC are immunostained to visualize F-actin filaments (green), and nucleus (blue). H441 cells appear round on both fiber meshes, however, HUVEC appear elongated and occupy larger area on the porous surface fiber meshes; n = 115. Confocal images were also taken to visualize HUVEC on (**I**) smooth surface fiber meshes and (**J**) porous surface fiber meshes for focal adhesion vinculin (red) and nucleus (blue). The dotted boxes in (**I**), (**J**) represent mature vinculin on the porous compared to weak intensity points by cells on smooth fiber surfaces. (**K**) Using ImageJ, the fluorescence intensity of vinculin was quantified. Higher intensity of vinculin was displayed by cells on the porous surface fibers; n = 25. The line in the middle of the box denotes the median and the whiskers denote the minimum and maximum values. Scale bar: 20 µm.

To evaluate the effect of the secondary topographical cues on growth rate, proliferation assays were conducted, as seen in Fig. 2A,E. HUVEC and H441 cell growth was supported on both fiber surfaces, however, an increase in proliferation on day 3 was observed on the porous fiber surfaces. This can be attributed to the increased focal adhesion and interaction on porous surfaces that favors cell attachment and has been reported previously as well^43,53^. Although, with increasing culture time, the rate of proliferation was similar on both the fiber surfaces as analyzed on day 9. This is most likely due to attainment of confluency with increasing culture period on both the smooth and porous fiber surface. Additionally, single cell morphology was investigated, where the H441 cells did not display any significant difference in cell area and were characteristically round shaped on both the smooth and porous surface fibers, Fig. 2B–D. However, endothelial cells after 24 h, displayed significant difference in morphology in terms of cell spread area. HUVEC displayed a lower cell area on smooth surface fibers compared to a larger cell area and more elongated morphology on porous surface fibers, Fig. 2F–H. Moreover, cell nucleus was also analyzed using ImageJ where no difference was observed in nuclei area, although both the cells displayed a higher nuclear aspect ratio on porous surface fibers (Supplement figure S5).

Cell attachment on substrates is due to focal adhesions, where clustered integrins internally interact with the actin cytoskeleton and externally can interact with materials^54^. Vinculin is a mature form of focal adhesion involved in cell–cell and cell–matrix interactions^55^. An increased amount of vinculin was displayed by HUVEC on porous fiber surfaces as compared to that on smooth fibers. This could be attributed to increased fiber surface area and attachment points presented by the fiber surface pores and thus a stronger cell–matrix interaction on porous fibers. Moreover, vinculin was distributed in the cytosol of the HUVEC in smooth fibers in contrast to porous fibers, where the vinculin was accumulated more on the cell periphery, Fig. 2I,J. The defined presence of vinculin at the end of actin filaments around secondary pores may result in the elongated cell shape compared to the more spread morphology on cells in smooth fiber surface^56^. The HUVEC are sensitive to the presence of pores on fiber surface by exhibiting elongated cytoskeletal morphology and increased and well-defined mature sites of vinculin, Fig. 2K. However, the H441 cells were not analyzed for vinculin as cancer cells are known to lack significant expression of vinculin^57^.

### Development of endothelial monolayers

**Figure 3:**
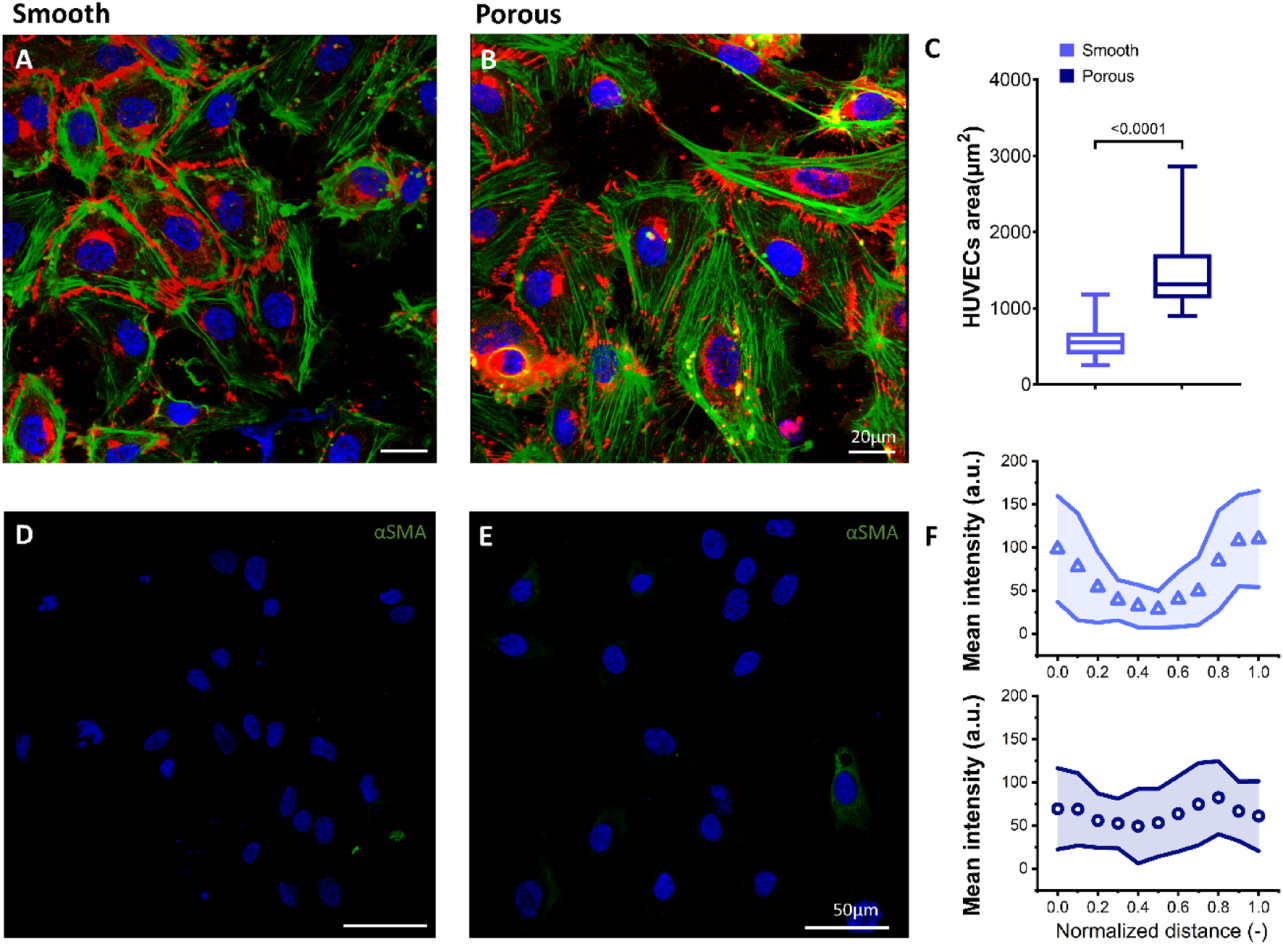
10-day culture of HUVEC endothelial monolayers on (**A**) smooth and (**B**) porous fiber surfaces were immune-stained to visualize F-actin filaments (green), cell–cell adhesion junction VE-cadherin (red) and nucleus (blue). Area occupied by individual cells in the monolayer was lower on smooth compared to that on porous surface fibers as seen in graph (**C**), where the line in the middle of the box denotes the median and the whiskers denotes the minimum and maximum values. The organization of F-actin filaments is majorly on the periphery of cells on the smooth fibers compared to the porous surface fibers where the actin filaments are more spread and depict stress fiber formation. Scale bar: 20 µm. Endothelial to mesenchymal transition is absent as cells did not stain positive for the mesenchymal marker αSMA on both (**D**) smooth and (**E**) porous surface fibers, (**F**) The actin profile was quantified for single cells along their long axis. n = 30. Scale bar: 50 µm.

Endothelial cell layer forms an important aspect of the alveolar-capillary barrier which is directly exposed to blood and is the first point of contact of the neutrophils during an immune response. Maintaining this integral layer prevents disruption of the alveolar function. Extensive research has been conducted on the behavior of confluent endothelial cells on electrospun nano- and micro-fibers both aligned as well as randomly arranged^40,41,44,58^. However, studies have rarely been conducted to understand behavior of endothelial monolayers on non-woven meshes displaying secondary topographical cues on fiber surface. The endothelial cells form monolayers on both the porous and smooth surface fibers as seen in Fig. 3A,B, where they display endothelial adhesion molecule VE-cadherin and F-actin filaments. However, a closer look at the cell morphology reveals that the endothelial cells are less spread on smooth surface fibers with characteristic display of actin filaments predominantly on the cell periphery, Fig. 3C. In comparison, the endothelial cells are more spread and display actin filaments throughout the cell body in the form of stress fibers on the porous fiber surface, Fig. 3F. It has been reported that a weak cell-substrate interaction leads to reduced cell spread and actin stress fiber formation in comparison to a more spread morphology with increased stress fiber formation displayed by strongly adhered cells^59–61^. Stress fibers are known to form during substrate interaction and formation of mature focal adhesions, which indicates and supports the interaction of cells with the fiber surface pores^62^. These observations are in accordance with single cells where increased amount of mature focal adhesion vinculin was observed on porous fiber surfaces suggesting a stronger cell–matrix interaction. Another important aspect of influence of secondary topographical features on endothelial to mesenchymal transition (EndMT) was also investigated. EndMT is the ability of endothelial cells via a cascade of molecular events to develop phenotypic features and transition into mesenchymal cells^63^. Upcoming developments of tissue engineered substrates by introduction of topographical cues have allowed improved cell adhesion and proliferation. However, this subject is not well studied and still lacks information regarding the cause and role of EndMT^64,65^. To understand whether addition of secondary topographical features on fiber surface would lead to EndMT, the endothelial monolayers were checked for mesenchymal marker αSMA by immunostaining. Day 10 cultures of endothelial cells on both smooth and porous meshes displayed no αSMA markers indicating that surface topography on electrospun fiber surface did not lead to EndMT, Fig. 3D&E.

### Development of epithelial monolayers

Alveolar barrier consists of alveolar epithelial type I & II cells, which together form an intact barrier due to stable cell–cell tight junctions. This layer is integral in secretion of surfactant proteins and preventing edema by remaining impermeable to major proteins and solutes^66^. Not many studies have been conducted to understand the role of secondary topographical cues in maintaining integral cell–cell junctions and formation of stable alveolar epithelial barriers^27,29,67^.

#### Effect of topography on barrier property

**Figure 4:**
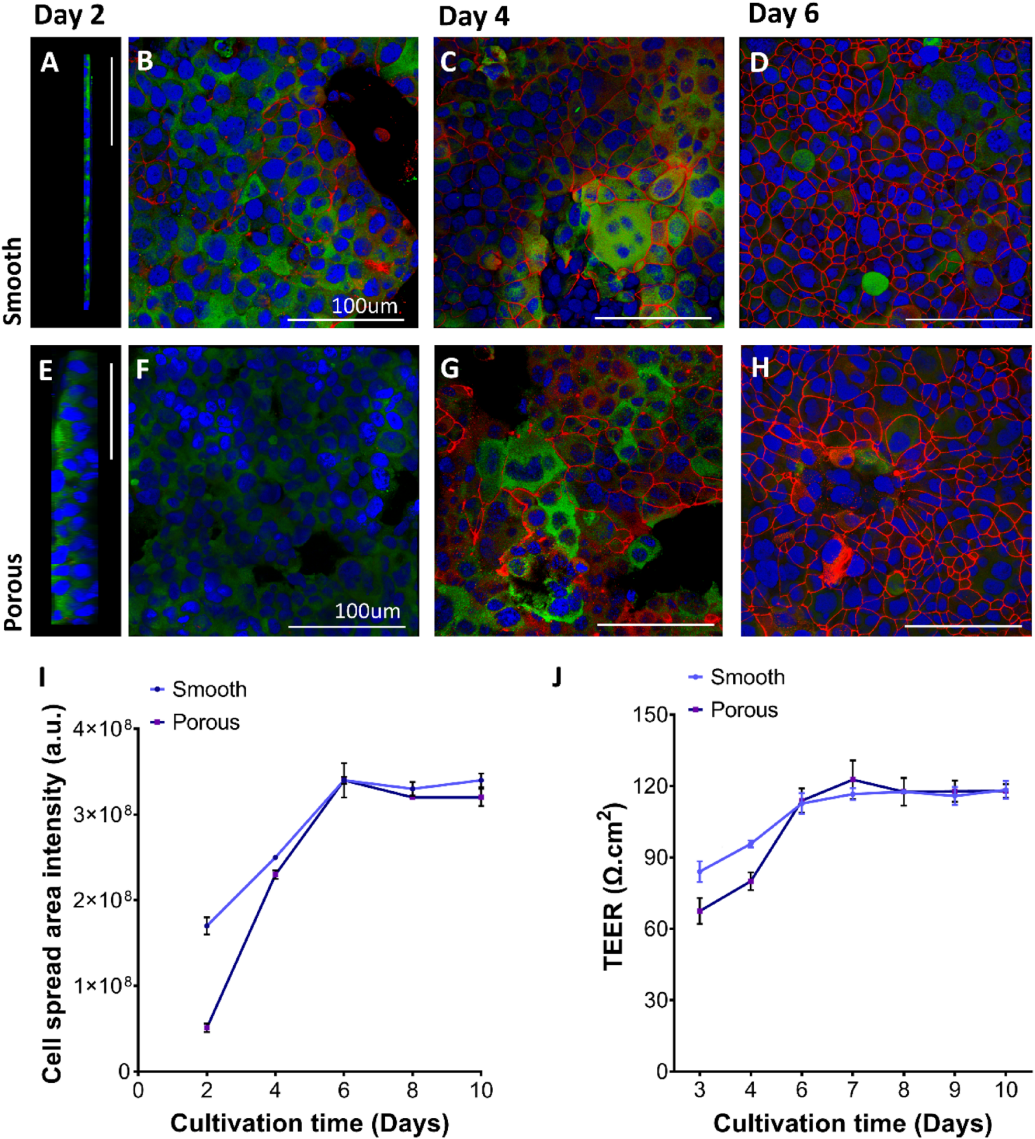
Integrity and spread of H441 epithelial cells was analyzed with increasing culture period to mark the time point at which tight junctions are formed as well as confluency is achieved. (**A**–**H**) H441 cells were immunostained for cell–cell tight junction ZO-1 (red), vinculin (green) and nucleus (blue). H441 cells appear to form (**A**) non-confluent monolayers on smooth surface fibers as seen in the cross-section view compared to (**E**) non-confluent multilayered formation on porous surface fibers. The initial tight junctions are formed already on (**B**–**D**) day 2 cultures of smooth fiber surfaces and tend to increase with time where cells reach confluent barrier after day 5, (**F**–**H**) but initial tight junction formation is only observed later on day 4 and attain confluent barriers after day 5 on porous surface fibers. This trend is supported by (**I**) cell spread area where H441 cells form multilayered island groups on porous surface fibers and monolayer islands on smooth surface fibers. The area increases with time as cells cover and spread more to form integral monolayers by day 6; (**J**) the barrier properties were analyzed quantitatively by periodic measurements of TEER as well where cells initially display higher values from day 2 onwards on smooth surface fibers. The values reach a plateau phase with culture time when cells reach confluency in both the cases. n = 3. Scale bar: 100 µm.

A quantitative and qualitative periodic investigation of epithelial (H441) monolayer formation was conducted to analyze the growth as well as formation of tight junctions with culture time. Initial growth pattern of H441 on day 2 was significantly different, where cells grew as multilayered islands on the porous surface fibers compared to non-confluent single layer on smooth surface fiber as seen in Fig. 4A,E. The formation of tight junctions (observed by ZO-1 immunostaining) was initially observed on smooth surface fibers from day 2 onwards, as seen in Fig. 4B–D. However, with increasing culture time and addition of dexamethasone (day 3), confluent monolayers were also achieved on porous surface fibers from day 5 onwards, Fig. 4F–H. This is also observed in the cell spread area, Fig. 4I, occupied by H441 cells with increasing culture time. The cells occupy more area on smooth surface (Supplement figure S6), corresponding to a monolayer development and less area on porous fibers despite the high rate of proliferation that represents a multilayer development. However, from day 5 onwards the cells were confluent, and the addition of dexamethasone (day 3) inhibited multiple layer formation as reported previously^68^. This is supported by a similar trend observed via TEER (Transepithelial electrical resistance) measurements, Fig. 4J, where higher values are observed on day 2 and 4 cultures on smooth fiber surface. These values reach a plateau from day 5 and were not significantly different suggesting attainment of confluent integral monolayers on both porous and smooth surface fiber with longer culture periods. Despite earlier development of tight junctions on smooth surface, the formation of confluent integral layers with increasing culture time is observed on both porous and smooth surface fibers.

#### Topography affects the microvilli and lamellar bodies

**Figure 5:**
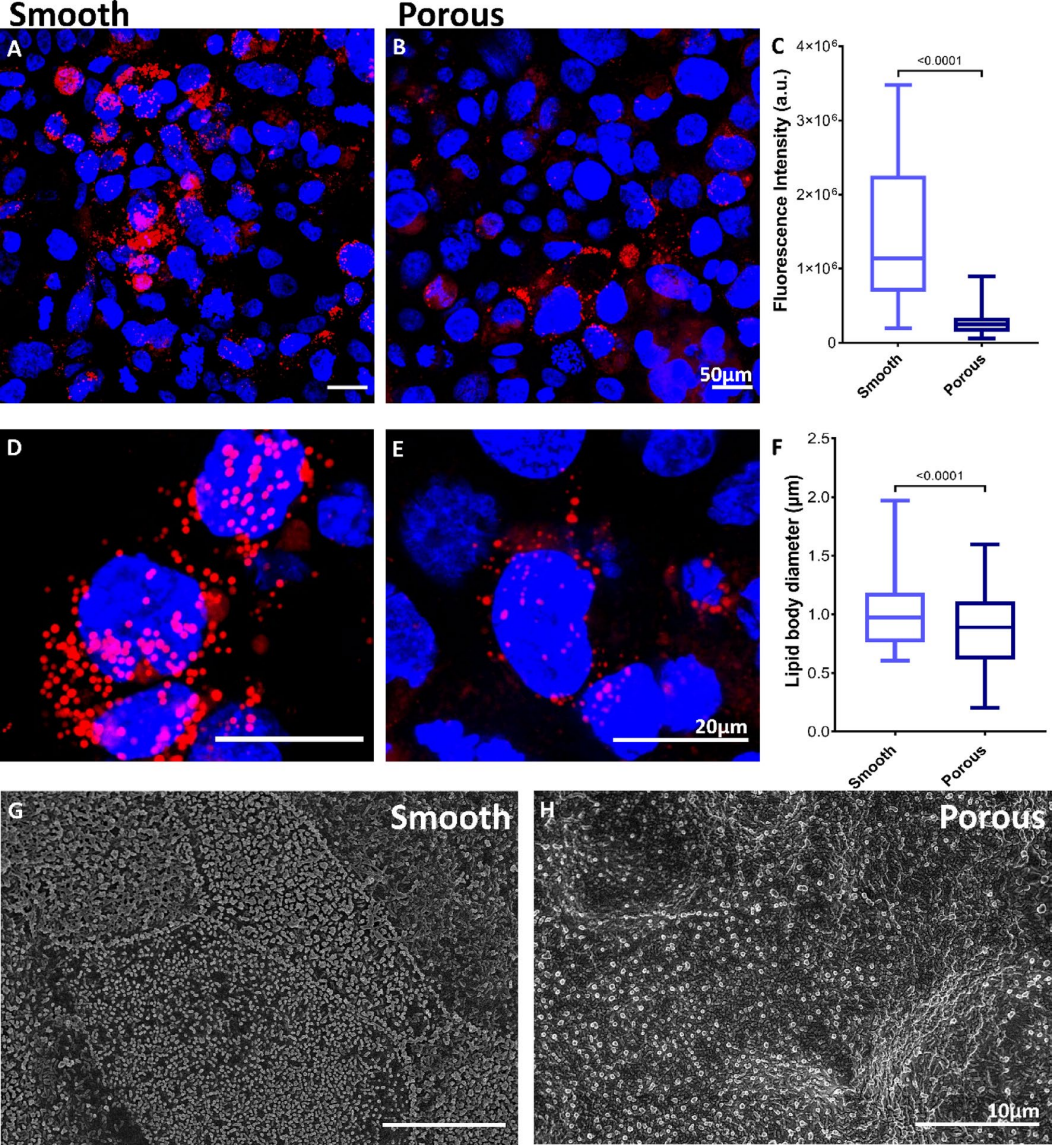
Monolayer H441 cells were analyzed for presence of characteristic properties of alveolar type II cells including lamellar bodies and microvilli formation. Day-10 H441 cells were stained to visualize lamellar bodies using Nile red on (**A**) smooth and (**B**) porous surface fibers, Scale bar: 50 µm, and quantitatively analyzed using ImageJ (**C**) where a significant increase was found on smooth surface fibers; n = 25. Closer view of the lamellar bodies is shown on (**D**) smooth and (**E**) porous surface, Scale bar: 20 µm, (**F**) This surfactant storing organelle was also quantified to have a diameter of nearly 1 µm in both the cases; n = 535. Microvilli, an important feature involved in increasing cell surface area was also well developed as seen on electron micrographs of (**G**) smooth than on (**H**) porous surface fibers. For box graphs (**C** and **F**), the line in the middle of the box denotes the median and the whiskers denotes the minimum and maximum values. Scale bar: 10 µm.

Alveolar type II cells are involved in synthesis and secretion of a complex mixture of phospholipids known as surfactant, which is stored in special organelles called lamellar bodies^69^. This special secretion of surfactant lowers surface tension and protects alveolar cell layer at the air–liquid interface from the mechanical stress and prevents alveolar collapse during breathing^70^. Additionally, alveolar type II cells are characterized by presence of microvilli that increases surface area for efficient gas exchange and absorption^71^. Therefore, influence of secondary topographical features on the development of H441 cells towards attaining similar phenotypical characteristic of alveolar type II cells was further investigated in terms of lamellar body and microvilli formation. This area has not been well investigated and is integral in developing stable and functional in vitro alveolar barriers. Lamellar bodies were visualized using nile red, a lipophilic dye used to stain lamellar bodies and surfactant lipids as well, Fig. 5A,B,D,E. We observed that the number of lamellar bodies were significantly higher in H441 cells on the smooth fibers as compared to the porous fibers, Fig. 5C. Additionally, the size of lamellar bodies was also quantified to be approximately 1 µm in diameter on both smooth and porous surface fibers, Fig. 5F. Although a large variation in lamellar body size was observed on smooth fibers compared to porous surface fibers, it is known to naturally range between 0.2 µm–2.4 µm in pulmonary alveolar cells^72^.

The cells were further analyzed for presence of microvilli on their surface using electron microscopy as observed in the Fig. 5G,H. Well-developed microvilli were found on epithelial cells cultured on smooth surfaced fibers in comparison to the cells present on fibers with surface pores. The secondary cues on fiber surface of non-woven mesh do not support functional alveolar epithelial cells, whereas the epithelial layers display characteristic properties of the alveolar type II cells in the absence of such secondary nano-cues. These observations indicate the use of smooth surface fibers to support stable and functional alveolar barriers.

## Conclusion

Development of stable and functional in vitro alveolar-capillary barriers is fundamental in the field of pulmonary tissue engineering. Here, we aim to elucidate the effect of secondary topographical cues on non-woven fiber mesh surfaces in the development of stable and functional in vitro alveolar-capillary barriers. Fibrous non-woven PCL scaffolds with secondary features were fabricated using electrospinning techniques to mimic the BM features. Our study reveals that the influence of the secondary cues varies between single cells and monolayers, thereby indicating a more complex relationship between cells and substrates that is currently known (Table 1). Additionally, nano-cues seem to have a significant influence on cell behavior compared to the micro topography which is similar in both the meshes. The single cell studies involving endothelial and epithelial cells were in accordance with previous literary works, where cells displayed predominant focal adhesions (vinculin) and higher proliferation rates on porous surface fibers compared to the smooth surface fibers. The nano-pores on the surface of micro-fibrous scaffolds provides additional anchorage points that improve interaction between cells and scaffolds^1,73^. Stable anchorage of adherent cells further promotes stronger cell-substrate interactions and enhances cell proliferation. However, a different behavior was displayed by the monolayer of cells where, the epithelial cell layers developed tight junctions as early as day 2 on smooth surface fibers compared to the uncharacteristic multiple cell layer formation on the porous surface fiber. Epithelium cell lineages are commonly used to study different types of cell adhesion including cell–matrix and cell–cell adhesion. Cell–cell adhesion is fundamental for development of organs and tissues and is mediated by tight junctions, adherens junctions and desmosomes. It has been shown that reduced expression of tight junction associated protein ZO-1 correlates with increased proliferation of epithelial cells^2,74^, which is seen in the case of porous surface fiber meshes. However, very little is known about the molecular basis and activated downstream pathways between ZO-1 and cell behavior. Additionally, the endothelial monolayers did not undergo EndMT transition on both types of fiber surfaces despite displaying lower actin stress formation on the smooth surfaced fibers. Furthermore, stable and functional alveolar barriers displaying features of alveolar type II cells were not obtained on porous surface fibers despite stronger cell-fiber adhesion. This can be attributed to stronger cell–cell interaction compared to cell-substrate interaction, which has also been observed in HUVEC by the group of Stroka et al., where experimentally weakened cell–cell junctions led to enhanced cell-substrate adhesion^75^. The H441 epithelial cell layers distinctly displayed phenotypical characteristics of alveolar type II cells including lamellar bodies and stable microvilli in the absence of topographical cues on fiber surface. It can be hypothesized that porous surface fibers provide a stronger cell–matrix association and weaker cell–cell adhesion in comparison to weaker cell–matrix and stronger cell–cell adhesion on smooth surface fibers. A stronger cell–cell adhesion activates molecular pathways for stable tight junctions, enhanced lamellar body and microvilli formation.

**Table 1:** Represents a comparative list of properties observed in the presence and absence of the secondary topographical cues on the fiber surface of the PCL meshes.

Primary topographical cues	Secondary topographical cues
Micro scale	Nano scale
Random fibrous architecture	Surface pores
Pore area 1.93 µm^2^ (Porous) and 1.30 µm^2^ (Smooth)	Pore area 0.01 µm^2^
–	Anchorage points for cells
–	Increased cell proliferation
Lower actin stress fibers	Significant actin stress fibers
Cytoplasmic vinculin	Vinculin in cell periphery
Stable alveolar barriers with microvilli and lamellar bodies	–

However, future investigations in terms of molecular gene expression in the presence and absence of secondary topographical cues on fiber surfaces could shed more light on such phenotypic expressions. Additionally, as an outlook in order to gain a deeper understanding of mechano-transduction via topological cues on cell morphology and behavior, future investigations using stem cells and the consequent YAP/TAZ analysis will be conducted. These findings could help determine the appropriate design of biomaterials suited for investigations and development of in vitro alveolar barrier models for physiological and pharmacological studies.

## Materials and methods

### Electrospinning

PCL (poly(caprolactone) M_n_ 80,000, M_w_/M_n_ < 2) (Sigma Aldrich, Germany) pellets were weighed and dissolved in 90:10 (v/v) chloroform (VWR, Germany) and DMSO (Dimethyl sulfoxide) (VWR, Germany) to obtain 12.5 wt/v% solution for porous surface fibers. Similarly, to obtain smooth surface fibers, PCL pellets were dissolved in 50:50 (v/v) chloroform and methanol (VWR, Germany) resulting in a 7.5 wt/v% solution. The electrospinning set-up consisted of an earthed spinneret as a syringe with a flat-tipped 27 gauge needle filled with the respective polymer solution. As a collector an aluminium foil of 20 cm × 20 cm was connected to a high voltage supply.

A high enough electric field to overcome the droplet surface tension was used to generate a taylor cone that elongated to form fibers and collected as non-woven mesh on the aluminium collector.

The spinning parameters were optimized to 21 kV, 17 cm (distance between collector and spinneret), and 0.75 mL/h to obtain homogenous non-woven fibrous meshes with pores on the fiber surface. Similarly, the spinning parameters to obtain homogenous non-woven meshes with smooth fibres were optimized to 21 kV, 10 cm and 1 mL/h. The time of electrospinning was constant at 2 min for 10 µm thin meshes in both the cases.

### Electrospun mesh characterisation

#### Fiber diameter and mesh pore size and fiber surface pores

The Electrospun meshes were prepared by sputtering a thin 10 nm layer of Au/Pd before imaging. Images of prepared electropsun meshes were captured using the S-4800 ultrahigh-resolution Scanning Electron Microscopy (SEM) from HITACHI, Japan. Images were taken at using an accelerating voltage of 20 kV setting and a working distance of 10 to 15 mm. These images were analyzed using ImageJ software for fiber diameter and pore area. Additionally, single fibers were analyzed to determine the size and area of the surface nano-pores using ImageJ. In brief, images were converted to 8 bits and subjected to thresholding to obtain clear features of the pores. The built-in feature of analyze particles was run to measure area, size, major and minor axis of the pores. Dye diffusion across the meshes was conducted via permeability assay using FITC-dextran 70 kDa (46 945 Sigma Aldrich, Germany). The meshes were fixed to inserts via O-rings, and 500 μL of 1xPBS was added to the basal chamber and 150 μL of 500 μg/mL of FITC-dextran was added to the apical chamber. 50μL of samples were collected from the basal chamber at different time intervals of 0, 5, 15, 25, 35, 45 and 60 min respectively. Every sample removal was replaced by additional 50 μL of 1xPBS. The fluorescent intensity of the samples was measured using Infinite M200 Tecan plate reader, Germany, at excitation wavelength 492 nm and emission wavelength 518 nm.

### Tensile stress and water contact angle measurements

The tensile properties of the porous and smooth meshes were studied using a 100N load in an AllroundLine, Zwick Roell (Germany). To enable attachment of the meshes to the tensile tester clamps, the meshes were electrospun on aluminum frames (2 cm × 1 cm). The frames along with the mesh were then attached to the clamps, where the edges of the frames were cut off to exclude interference in measurements by the frame, and tensile properties of only PCL mesh were measured. The first 10% of the linear strain region of the stress–strain curve was used to calculate the Young’s modulus.

The wetting properties of the non-woven meshes are analyzed by measuring static water contact angle with a sessile drop method using Krüss Drop shape analyzer (DSA100, Germany). Distilled water droplets of 3µL volume are placed randomly on the non-woven meshes and the contact angle is measured using the inbuilt software DSA4.

### 3D printed inserts and mesh attachment

The electrospun meshes were supported on 3D printed inserts made in-house, due to the physical adhesion of the hydrophobic meshes onto the hydrophobic inserts. This interaction prevents further treatments including usage of medical glue to attach the mesh on the insert. A prototype of the insert was designed using Autodesk inventor professional 2017, as in previous publication^27^. In brief, the inserts were printed by polyjet 3D printing (Stratasys, Germany, Objet Eden 260 V) using a mixture of two polymers. The combination of a photosensitive & acrylate-based (Stratasys, Germany, RGD810) polymer and support polymer (Stratasys, Germany, SUP705) was used, where after printing, 1 M NaOH was used to dissolve this supporting polymer. The meshes were carefully removed from the aluminum foils using forceps. The meshes were then immersed in 70% ethanol for sterilization followed by washing in PBS to remove ethanol residues. The meshes were then transferred onto the inserts followed by UV sterilization for 30 min on opposite sides each and shortly plasma treated for 15 s at 30Watts and 30 mL/min of oxygen flow rate. This was then coated with 100 µg/mL collagen I (C424 Sigma Aldrich) to initiate better cell adhesion, Supplement Figure S7.

### Cells and cell culture

Primary Human Umbilical Vein Endothelial Cells-HUVEC (Promocell, Germany) and Human lung adenocarcinoma cell line NCI-H441 (ATCC, America) were used. As growth medium, endothelial cell growth media (C-22111) were purchased from Promocell and for H441 cells RPMI-1640 (Thermofischer Scientific, Germany) was used. Penicillin/streptomycin, and trypsin were also obtained from Thermofischer Scientific including Fetal Bovine Serum-FBS (Biowest, Germany).

HUVEC was maintained in culture flasks with endothelial cell growth medium at 37 °C and 5% CO_2_. When cells achieved 85% confluency they were subcultured or used for further experiments. HUVEC, not more than passage 5 was used. The cell line NCI-H441 was cultured in RPMI-1640 supplemented with 1%penicillin/streptomycin and 10% FBS at 37 °C and 5% CO_2_. Subculture and further use in experiments were carried out when these cells were 80–85% confluent. NCI-H441 cells below passage 40 were used for experiments.

For single cells studies, the prepared inserts with respective non-woven meshes were seeded with cell culture suspension of HUVEC and H441 at 7.5 × 10^4^ cells/cm^2^. After 24 h, the single cell cultivated samples were fixed and further analysed using immunohistochemistry. Monolayer cultivation and cell proliferation XTT assays were conducted by seeding cell suspensions each of HUVEC and H441 at 4.5 × 10^5^ cells/cm^2^ on respective non-woven meshes.

### XTT cell proliferation assay

To determine rate of proliferation on the fabricated non-woven meshes, XTT proliferation kit was purchased from ATCC, America. The proliferation of both HUVEC and H441 on respective meshes was analyzed separately on day 3 and 9. The membranes were washed with 1xPBS before adding the XTT reagent and activation reagent at (10:1) provided in the kit. 100µL of the activation and XTT reagent was mixed with 50 µL of phenol free medium. This 150µL was added to each insert and was incubated for 3 h. The tetrazolium XTT dye is orange in color and is reduced to a brightly colored formazan derivative in the presence of viable cells. This reduction is due to the electrons at the plasma membrane of cells with active mitochondrial activity. This bright orange dye is quantified and detected by absorbance measurements using a spectrophotometer (SpectraMax M3, Molecular devices, Germany) at 490 nm and 630 nm respectively. Additionally, to eliminate background from the media, absorbance of blank wells without cells is measured. The final absorbance is calculated by subtracting the respective values with average of the blank absorbance.

### Immunocytochemistry

Prior to staining, 4% paraformaldehyde at room temperature was used to fix the cells on meshes for 15 min. To enhance dye uptake, permeabilization with 0.25% Triton-X (Sigma Aldrich, Germany) for 10 min was conducted. This was followed by blocking with 3% BSA for 60 min to prevent non-specific adsorption. The actin cytoskeleton was stained for F-actin by incubating the samples in Phalloidin iFlour 488 reagent (1:1000 Abcam, Germany) for 2 h. The mature focal adhesion vinculin was stained using primary antibody vinculin (1:100 Invitrogen, Germany) for 2 h followed by PBS wash. Secondary antibody goat anti-mouse Alexa 594-conjugated IgG (1:200 Invitrogen, Germany) was added for 2 h followed by PBS wash. To visualize the cell–cell junctions of HUVEC, the samples were incubated with primary antibody VE-cadherin (1:100 Santa cruz biotechnology, Germany) for 2 h followed by PBS wash. Secondary antibody goat anti-mouse Alexa Flour 488-conjugated IgG (1:200 Abcam, Germany) was added for 2 h followed by washing with PBS. To visualize tight junctions and mesenchymal αSMA, the samples were incubated with primary ZO-1 antibody (1:100 Thermofischer Scientific) or α-SMA (1:50 Sigma Aldrich, Germany) at 4 °C overnight. The next day, the addition of secondary goat anti-rabbit Alexa Flour 594 conjugated IgG (1:200 Abcam, Germany) or secondary goat anti-mouse Alexa Flour 488 conjugated IgG (1:200 Abcam, Germany) for 2 h. This was followed by PBS wash and addition of DAPI (1:1000 Molecular probes, Germany) to counterstain the nuclei with subsequent PBS washing steps. All the PBS washing steps included 3X wash for 5 min each. The lamellar bodies were stained using Nile red reconstituted in 100% ethanol at 1 mg/mL (1:100 Sigma Aldrich Germany) for 2 h. After staining, the samples were finally cut-off from the inserts using a surgical grade scalpel (Braun) and observed with Confocal Laser Scanning Microscope (CLSM Leica TCS SPE, Leica, Germany).

### Barrier integrity using trans-epithelial electrical resistance (TEER)

The epithelial monolayer on smooth and porous surface fibers were analyzed for tight junction formation or integral barrier formation using the EVOM2 Epithelial Tissue Volt/Ohmmeter from World Precision Instruments (Germany). The mesh attached to the insert allowed measurement using the standard STX2 chopstick electrode. One electrode arm was placed in the lower well and the other in the upper well to measure the resistance across the cell layer. Additionally, resistance across blank meshes without cell layers were measured and subtracted from the resistance (Ω) measured across the cell layer. This difference in value was further multiplied by the cell growth area (0.33 cm^2^) to evaluate the TEER (Ω cm^2^) across epithelial monolayer.

### Electron microscopy

Field Emission Scanning Electron Microscope (FESEM) was performed using S-4800 ultra-high-resolution SEM (HITACHI, Japan) to analyze fiber characteristics of electrospun meshes as well as cell morphology at an accelerating voltage (20 kV) and a working distance (10–15 mm). The meshes with fixed cells following 4% paraformaldehyde treatment, were further treated to undergo a series of drying steps that involved using ethanol (30%, 50%, 70%, and 100%) for 10 min each. This was followed by drying using Hexamethyldisilazane (Sigma Aldrich, Germany) for another 10 min. The dried samples were then sputtered with 6 nm layer of Au/Pd and used for analysis.

### Statistical analysis

All data analyzed are expressed as mean ± standard deviation, unless stated otherwise in the figure captions. Three individual experiments were carried out for statistical analysis, unless stated otherwise in the figure captions. The groups were analyzed for statistical differences in Graphpad Prism v9 software by using the unpaired T-test with Welch’s correction. Data groups representing TEER and cell spread area were analyzed with two-way Anova and tukey’s mean comparison. An alpha value p less than 0.05 was considered statistically significant. The p values are indicated in the respective graphs.

## Supplementary Information

Supplementary Information 1.

## Data Availability

The datasets generated during and/or analysed during the current study are available from the corresponding author on reasonable request.
