# Supplementary material for: Topographical influence of electrospun basement membrane mimics on formation of cellular monolayer
Source: Sci Rep. 2023 May 24;13:8382. doi: 10.1038/s41598-023-34934-x (PMC10209110; doi:10.1038/s41598-023-34934-x)
Supplement: Supplementary file 1 — Supplementary Information 1. [file 41598_2023_34934_MOESM1_ESM.pdf]

Supporting Information

Topographical influence of electrospun basement membrane mimics on formation of cellular monolayer

Puja Jain[1,#], Rahul Rimal[1,2,#], Martin Möller[1], Smriti Singh[2,]*

[1] DWI-Leibniz Institute for Interactive Materials, Forckenbeckstr. 50, 52056 Aachen, Germany
[2] Max Planck Institute for Medical Research (MPImF), Jahnstrasse 29, 69120 Heidelberg, Germany
[*] Corresponding author, Email address: smriti.singh@mr.mpg.de
[#] authors contributed equally to work

Water Contact Angle

The wettability of the fibers as well as influence of surface nanopores were measured using sessile drop method. 3µL volume of distilled water drops are placed randomly on the non-woven meshes and the contact angle is measured using the inbuilt software DSA4. Non-woven meshes with pores on fiber surface were $131.73^0 \pm 3.34^0$, which is similar to that measured on smooth fiber surface meshes $125.88^0 \pm 3.46^0$. This is due to the hydrophobic nature of PCL as well as the influence of inter-fiber distance of the non-woven mesh that predominates the effect of nanopores on fiber surface.

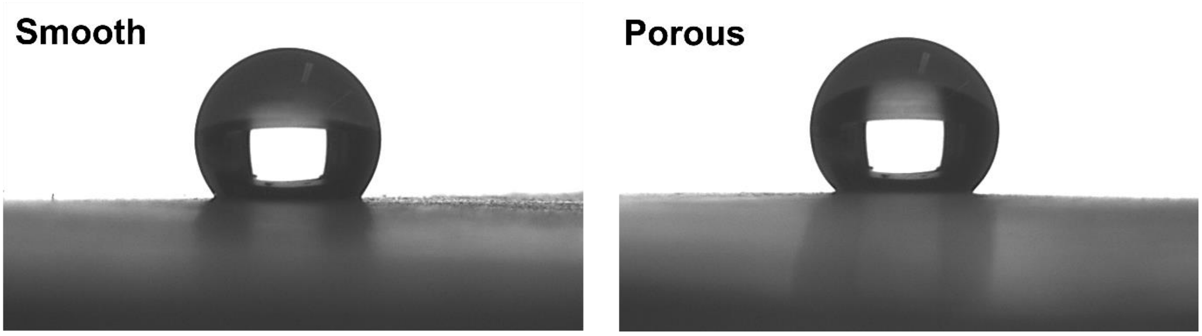

Figure S1. Images display the 3µL water drops on respective non-woven meshes with smooth and porous surface fibers with similar fiber diameter. Both the meshes display a stark hydrophobic nature which is an inherent property of PCL meshes.

Electrospinning parameters

PCL was dissolved in different combinations of solvents to fabricate smooth and porous surface fibers of similar diameter. PCL (w/v%) was dissolved in varying combinations of solvent systems as depicted in table below (Table S1). Additionally, the electrospinning parameters were varied in terms of the distance between collector and spinneret, voltage supply and the flow rate as well to obtain non-woven fibers of similar diameter but with and without surface pores. Other parameters such as room temperature and humidity were maintained to be constant.

Table S2. Represents a list of optimization parameters including flow rate (mL/h), distance (cm) and solvent system, to fabricate non-woven PCL meshes with and without surface pores, obtained in a vertical electrospinning device.

Flow rate [mL/h]	distance [cm]	Voltage [kV]	Solvent 1 [v %]	Solvent 2 [v %]	PCL [% w/v]	Type	Nature	Image
1	10	21	HFIP 90%	DMSO 10%	12.5	Smooth	Homogenous, Nano-fibers	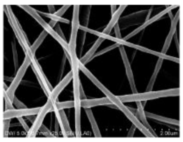
1	22	21	CF 50%	Methanol 50%	12.5	Smooth	Inhomogeneous	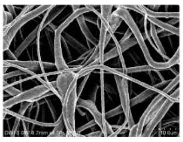
1	10	21	CF 50%	Methanol 50%	12.5	Smooth	Homogeneous, micro-fibers	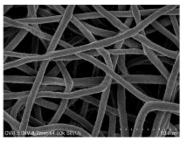
1	20	21	CF 90%	DMSO 10%	10	Porous	Beaded fibers	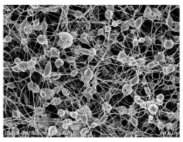
0.5	20	21	CF 90%	DMSO 10%	12.5	Porous	Glued fibers	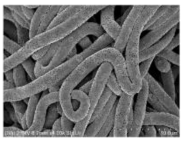
0.75	17	21	CF 90%	DMSO 10%	12.5	Porous	Homogenous, micro-fibers	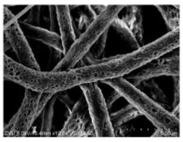

Permeability of FITC-dextran (70kDa) across the meshes

The diffusion of FITC-dextran (70kDa) from the apical side of the insert to the basal side was slightly more (as observed by the fluorescent intensity values of solution taken from the basal side) across the smooth meshes as compared to the porous meshes. However, there is no significant difference in passage of molecules across the smooth and porous meshes as they share a similar interfiber distance.

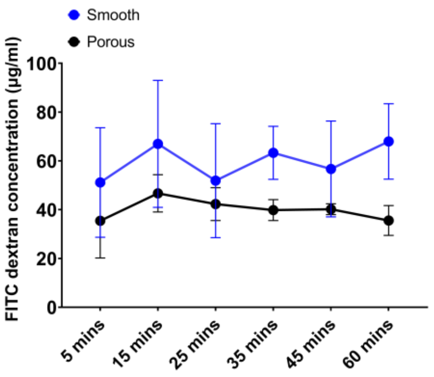

Figure S2. The graph represents diffusion of FITC-dextran (70kDa) across smooth(blue) and porous(black) meshes from 0 to 60 minutes. The smooth meshes display a slightly higher diffusion compared to porous surface fibers, despite similar interfiber distance.

Tensile Measurements

Tensile tester AllroundLine, Zwick Roell (Germany) was used to study determine elastic properties of the non-woven PCL meshes under the applied force of 100N cell load, where the Elastic moduli was determined by calculating the slope of stress over strain within 10% strain region. The figure displays measurement of triplicate samples of both smooth and porous surface fibers. It was observed that that greater stress was required for smooth surface fibers compared to the porous surface fibers. This is attributed to the nanopores on fiber surface that contribute as pre-existing fracture points, where smaller force is enough to induce a larger strain. This explains a higher tensile modulus for smooth (15.73MPa±3.55MPa) compared to the porous fiber meshes (5.47MPa±1.72MPa).

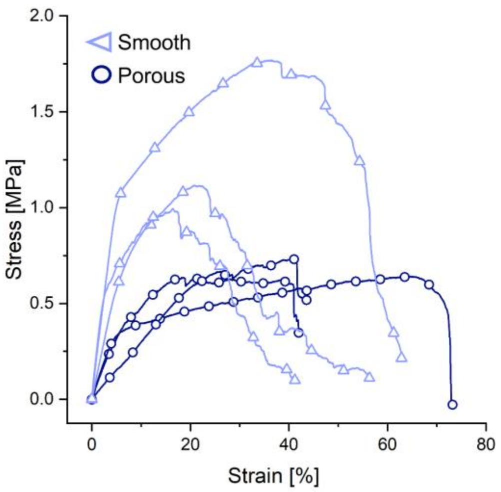

Figure S3. The graph represents the elastic property of both the smooth surface fiber and porous surface fiber non-woven PCL meshes under 100N load. More force is required for smooth surface fibers that have a higher tensile modulus compared to lower force required by porous surface fibers; n=3.

Single cell Analysis

Cells sense their microenvironment by membrane protrusions such as lamellipodia and filopodia in the initial stages of cell-surface interaction. In the case of porous surface fiber meshes, the cells extend their filopodia to feel the surface pores but do not seem to penetrate the surface pores. However, the cells are observed to interact with the surface pores via membrane extensions.

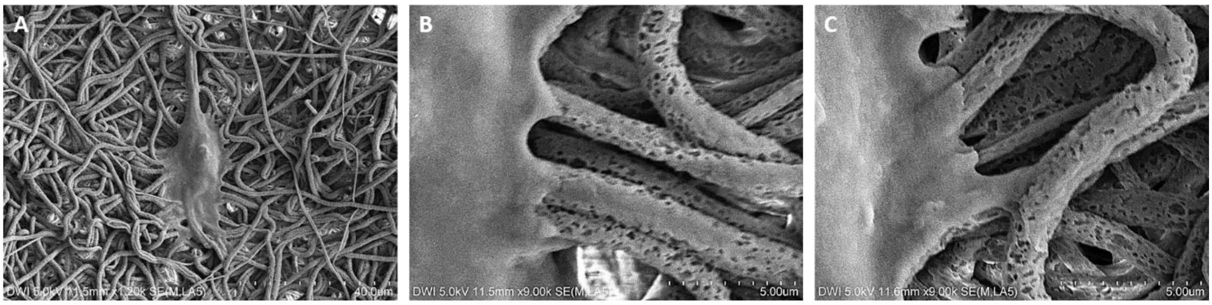

Figure S4. Single epithelial (H441) cell present on non-woven meshes of PCL with fiber surface pores is seen to interact via membrane extensions, where nano-pores serve as anchorage points.

The nucleus of single endothelial (HUVEC) and epithelial (H441) cells was also investigated using ImageJ analysis in terms of nucleus area and aspect ratio. The area of nuclei in both HUVEC and H441 cells did not show any difference on either the smooth or porous surface fiber meshes. However, as expected the aspect ratio of nuclei on porous surface fibers was higher than that on smooth surface fibers which is according to elongation of the HUVEC on porous surface fibers. However, we did not see any elongation of H441 cells themselves , but the nuclei were surprisingly affected by the nanopores and displayed a higher nuclei aspect ratio on the porous surface fiber meshes.

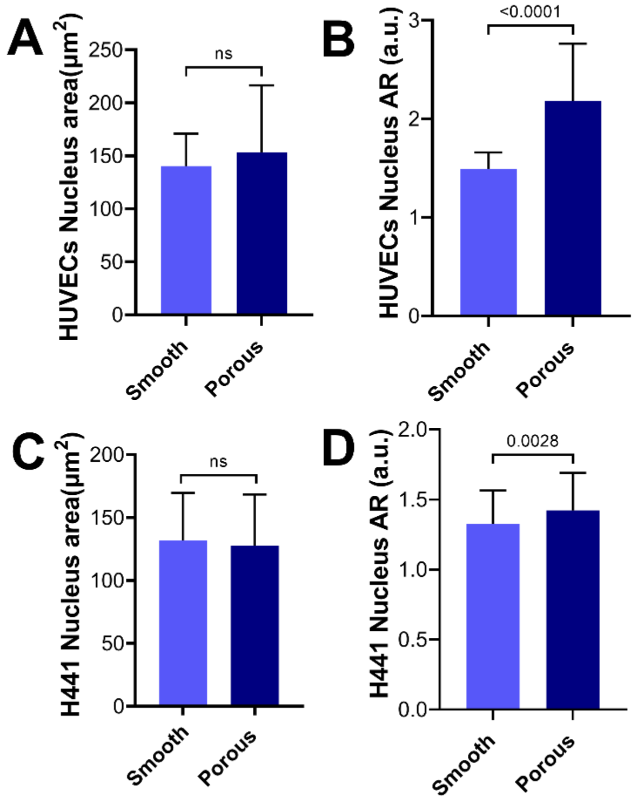

Figure S5. The graph represents the A), C) nuclei area and B),D) aspect ratio of respective single endothelial (HUVEC) and epithelial (H441) cells present on non-woven meshes of PCL with and without fiber surface pores. No difference was observed in nuclei area of both cells on the different meshes, but cells on porous surface fibers displayed a greater aspect ratio which implied nuclear elongation; n= 70.

Cell spread area of H441 monolayers

Raster images using confocal microscopy were taken to investigate the growth of H441 cells during the culture period from day 2 to day 10 on both the smooth and porous surface fiber meshes. The cells were stained for actin in green and the blue dye represents the DAPI stain for nucleus. On day 2 the cells are not confluent on both the porous and smooth surface fibers. However, the cells on the smooth surface fibers attain confluency on day 4 but gaps are still observed on the corresponding porous surface fiber meshes. Cells spread slowly on the porous surface fibers and thus take longer to attain tight barriers compared to when seeded on smooth surface fiber meshes.

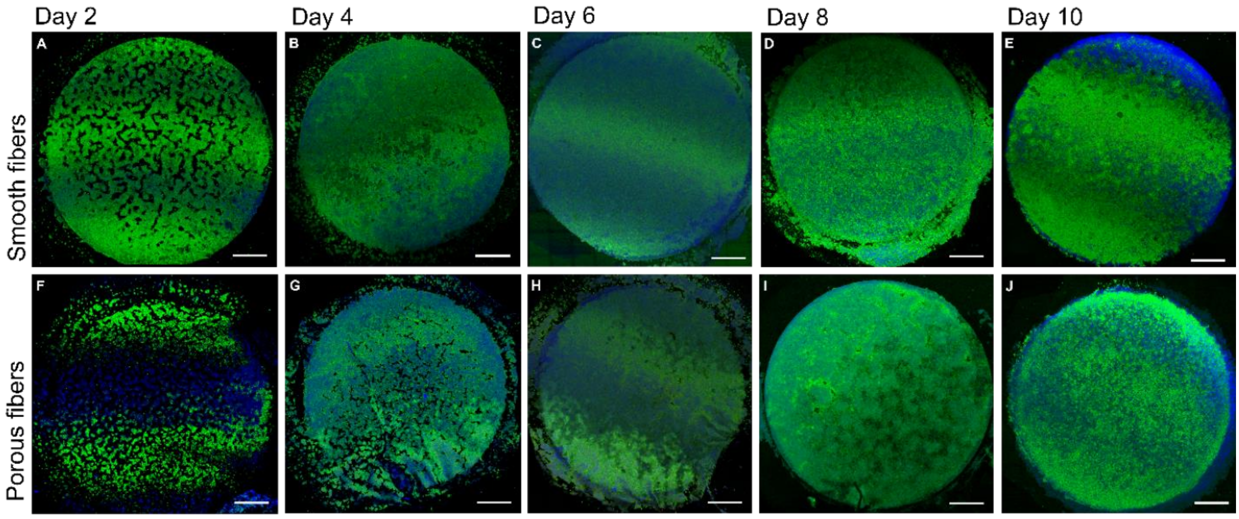

Figure S6. Raster confocal images of growth and spread of H441 cells on respective Smooth and porous surface fiber meshes. Cells obtain confluency on smooth surface fibers faster than on the porous surface fibers, where cells are immune stained for actin (green) and nucleus (blue). Scale bar: 1mm.

Schematic of mesh preparation for cell seeding

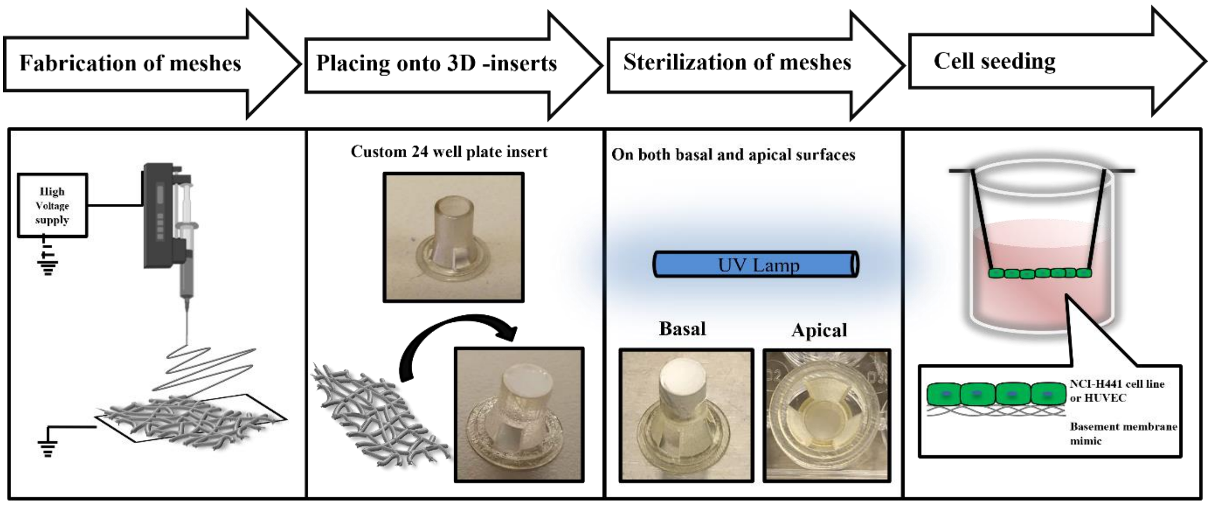

Figure S7. A general scheme that represents various steps conducted from mesh fabrication to cell seeding. The electrospun meshes obtained are transferred onto 3D inserts for 24 well plates, followed by sterilization using UV for 30 minutes each on both apical and basal side of the mesh. After sterilization, respective HUVEC or H441 cells are seeded on the meshes.
